# Clear Corneal Phacovitrectomy with Posterior Capsulorhexis and IOL Implantation in Management of Selective Vitreoretinal Cases

**DOI:** 10.1155/2015/474072

**Published:** 2015-10-26

**Authors:** Ernest V. Boiko, Sergey V. Churashov, Alexei N. Kulikov, Dmitrii S. Maltsev

**Affiliations:** ^1^St. Petersburg Branch of the Academician S. Fyodorov IRTC “Eye Microsurgery”, 21 Yaroslav Gashek Street, St. Petersburg 192283, Russia; ^2^Department of Ophthalmology, Military Medical Academy, 5 Klinicheskaya Street, St. Petersburg 194044, Russia

## Abstract

*Purpose*. To describe our technique, clear corneal phacovitrectomy with posterior capsulorhexis (CCPV), for the management of selected posterior segment intraocular foreign body (IOFB), posteriorly dislocated lens fragments (PDLF), and proliferative diabetic retinopathy (PDR) cases. *Methods*. This was a single-center retrospective interventional case series. In 21 patients (21 eyes) we performed phacovitrectomy through three clear corneal tunnel incisions (CCTI) and posterior capsulorhexis to remove IOFB (*n* = 8), PDLF from the vitreous cavity after complicated phacoemulsification (*n* = 6), and vitreous hemorrhage and epiretinal membranes in PDR (*n* = 7). The procedure was completed with implantation of a hydrophobic acrylic IOL through the CCTI. *Results*. The mean visual acuity (logMAR) was 0.90 preoperative and improved to 0.26 over a mean follow-up of 8.7 months (range, 6–12 months). The intraocular lens was implanted into the capsular bag (*n* = 12) or onto the anterior capsule (*n* = 9). One PDR patient experienced an intraprocedural complication, hemorrhage from isolated fibrovascular adhesions. One IOFB patient developed apparent anterior proliferative vitreoretinopathy and required a repeat intervention. *Conclusion*. Selected vitreoretinal IOFB, PDLF, and PDR cases can be successfully managed by a combined surgical approach involving clear corneal phacovitrectomy with posterior capsulorhexis and implantation of an IOL, with good visual outcome and a low complication rate.

## 1. Introduction

The advent of minimally invasive technique has changed the surgical approaches to management of both anterior and posterior segment disorders and provided the potential for performance of multiple surgical procedures in all ocular structures within the same operation, with prompt switching from one of the numerous entry sites and/or instruments to another. It is especially helpful in an open-globe injury (OGI), where a variety of alternating anterior and posterior segment maneuvers “pole to pole surgery” within the same operation are often required [1]. To deliver the best possible surgical outcome in a short time, the surgeon is to be skilled in both anterior segment (e.g., cataract and iris) and vitreoretinal surgery (VRS). Such a requirement occurs not only in ocular traumas (particularly intraocular foreign body (IOFB) injuries [2–5]), but also, for example, when the surgeon has to remove lens fragments from the fundus after complicated phacoemulsification surgery [6, 7] or to repair alterations in the central vitreoretinal interface in proliferative diabetic retinopathy (PDR) [8–10].

Therefore, the aim of this study was to describe our combined surgical approach involving clear corneal phacovitrectomy with posterior capsulorhexis (CCPV) and implantation of an IOL, for the management of selected posterior segment IOFB, posteriorly dislocated lens fragments (PDLF), and PDR cases.

## 2. Material and Methods

This study was conducted at Military Medical Academy (St. Petersburg, Russia) during 2010 to 2014. The study was approved by Ethics Committee of Military Medical Academy.

We retrospectively analyzed the surgical outcomes in 21 eyes of 21 patients who underwent CCPV for removal of 2 to 5-mm IOFB in type C OGI (*n* = 8) (according to the International Society of Ocular Trauma (ISOT) classification) [11, 12], lens nuclear fragments and lenticular matter from the vitreous cavity after complicated phacoemulsification surgery (*n* = 6), and vitreous hemorrhage in PDR (*n* = 7).

The main inclusion criterion for the procedure was the central vitreous cavity location and/or central fundus location of either IOFB or critical pathologically altered structures to be removed. The patients had had lens damage or opacities requiring phacoemulsification surgery (*n* = 15) or aphakia after complicated phacoemulsification surgery (*n* = 6). All of them underwent operation at our institution.

Retrospectively, the following characteristics were assessed: (1) preoperative, early postoperative (within 7 days), and final postoperative (6–12 months) visual acuity levels; (2) the potential to achieve the surgical goals: removal of cataract, IOFB, lens fragments, vitreous hemorrhage and proliferative tissue, and implantation of an IOL; (3) the occurrence of any of the following complications: (a) critical complications making the surgeon change the surgery plan, (b) noncritical complications hampering the procedure, and (c) late follow-up complications; (4) intraoperational pupillary changes; (5) the state of posterior capsulorhexis depending on its size and shape; (6) the potential to implant an IOL into the capsular bag or onto the anterior capsule; and (7) self-sealing characteristics of the incisions in the CCPV.

### 2.1. Preoperative Evaluation

Preoperatively, routine examination and, if indicated, ultrasound B-scan, roentgen, or computer tomography imaging were performed to localize IOFBs. Visual acuity was assessed using the Snellen Chart, and the results were converted to logMAR visual acuity for analysis.

### 2.2. Surgical Technique

The Infiniti Vision System (Alcon Laboratories Inc., Fort Worth, TX) and Accurus Vitrectomy System (Alcon Laboratories Inc.) were used to perform phacoemulsification of the lens and 25G+ vitrectomy, respectively.

#### 2.2.1. Phaco Surgery Stage

First, three clear corneal tunnel incisions (CCTI) were made in a standard manner in the superonasal, superotemporal, and inferotemporal quadrants (if the phacoemulsification stage was present, one 2.2-mm superonasal or superotemporal and two 1.0-mm incisions were made; if not, all the incisions were as wide as 1.0 mm). Phacoemulsification or, in eyes with soft nucleus (*n* = 5), phacoaspiration was then performed. In 15 cases undergoing planned phacovitrectomy, to secure the capsular bag and prevent iatrogenic damage to it, a capsular tension ring was implanted after performance of an anterior capsulorhexis with the diameter reduced to 4.5 mm.

#### 2.2.2. Vitrectomy

**Figure 1:**
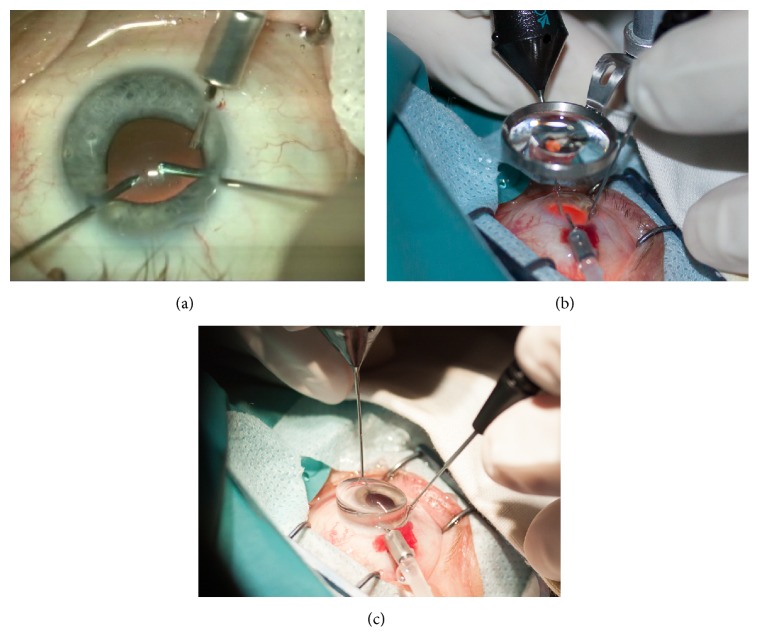
Intraoperative photographs showing the positions of the instruments at different stages of CCPV. (a) Initial stage (making the posterior capsulorhexis). (b) Use of BIOM 3 system with SDI II m invertor for posterior segment work. (c) Use of 8-mm diameter lenses of Pediatric Vitrectomy Lens Set.

In 6 cases undergoing planned phacoemulsification only surgery, it was converted to phacovitrectomy after damage to the posterior capsule and dislocation of nuclear fragments smaller than one-fourth the size of the lens nucleus to the vitreous cavity. Before the vitrectomy, an infusion cannula was inserted through the inferotemporal CCTI. 25G+ light probe and vitreous cutter were introduced through the original CCTIs in both superior quadrants (Figure 1(a)), and the cutter was used to make a posterior circular (3.5–4.0-mm diameter, *n* = 6) or oval (3.5 mm × 6.0 mm dimensions, *n* = 9) capsulorhexis. Vitrectomy was thereafter performed. During vitrectomy in OGI with IOFB, special care was given to the sites of vitreous strand attachment, retinal injury, and IOFB occurrence. A BIOM 3 system (Oculus, Wetzlar, Germany) with SDI II m invertor (Oculus) (Figure 1(b)) and 8-mm diameter lenses of Pediatric Vitrectomy Lens Set (Ocular Instruments Inc., Bellevue, WA) (Figure 1(c)) were used for the posterior segment work. The posterior hyaloid was removed in each patient. If there was no posterior vitreous detachment, triamcinolone acetonide (40 mg/1 mL) was injected for better visualization of the cortical vitreous. In two cases with IOFB, a cohesive ophthalmic viscosurgical device (OVD), Provisc (Alcon Laboratories Inc.), was injected intraoperatively to stabilize and protect the retina. A viscous dispersive OVD, DisCoVisc (Alcon Laboratories Inc.), was used to stabilize the anterior chamber and capsular bag and protect the corneal endothelium [13].

#### 2.2.3. Removal of Posterior Segment IOFB in Type C OGI

**Figure 2:**
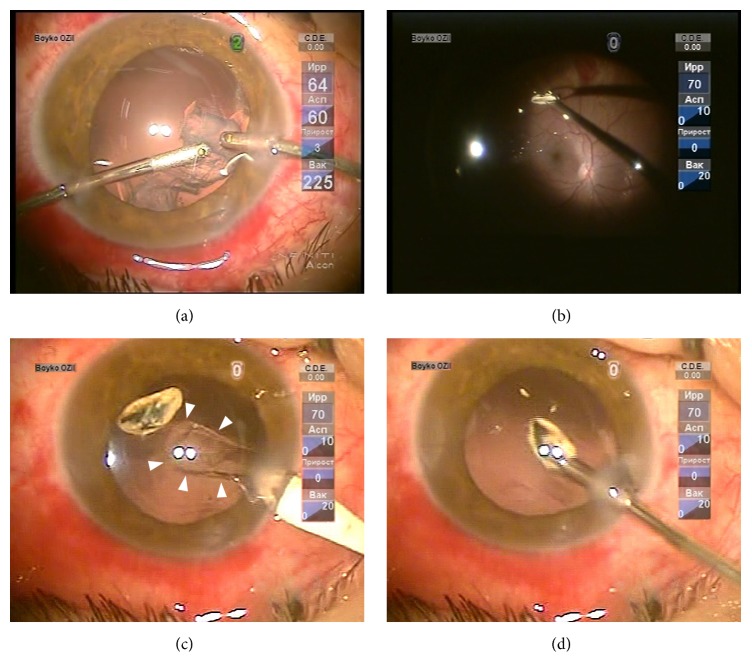
Intraoperative photographs showing the steps of CCPV in the patient with IOFB and traumatic cataract. (a) Completion of phacoemulsification: aspiration and irrigation of the lens material. (b) After vitrectomy, the IOFB was grasped with gripping forceps introduced through the clear corneal tunnel incision. (c) The IOFB was left on the posterior capsule of the lens for the time required to widen the clear corneal tunnel incision. The anterior chamber was filled with a cohesive ophthalmic viscosurgical device. Note the well-defined contour of the posterior capsulorhexis (arrowheads). (d) IOFB removal through the clear corneal tunnel incision of adequate width.

After completion of the phacoemulsification (Figure 2(a)) and vitrectomy, the foreign body, if any, was grasped with forceps (*n* = 6) (Figure 2(b)) or a magnet tip (*n* = 2) introduced through the CCTI and posterior capsulorhexis. If successfully grasped with forceps, the IOFB was delivered to the anterior chamber.

In 4/8 of IOFB cases, the IOFB was temporarily left on either the iris or anterior/posterior lens capsule (Figure 2(c)), outside of the capsulorhexis, enabling the surgeon to choose the most suitable forceps and incision width for its successful removal (Figure 2(d)) after removal of the BIOM 3 (from optical microscope system) or corneal lens. When a magnet was required to grasp a large IOFB, the vitreous cavity was filled with perfluorocarbon liquid (PFCL) to maintain the eye shape and immobilize the retina, and this IOFB was removed immediately through capsulorhexes and the CCTI. The PFCL was then easily removed from the eye through the original CCTI by aspiration. At the IOFB site and retinal injury site, barrier laser photocoagulation of the retina was performed when required (*n* = 4 and *n* = 2, resp.).

#### 2.2.4. Removal of PDLF from the Vitreous Cavity after Complicated Phacoemulsification Surgery

To remove lens nuclear fragments from the fundus, only core vitrectomy, without thorough surgical manipulations of the peripheral vitreous, was performed. The main goal of vitrectomy in those eyes was to prevent anterior chamber vitreous prolapse and vitreous incarceration in the CCTI wounds. Additionally, the lens fragments usually retained in the central fundus were to be removed completely from the eye. If the retained lens fragments were large and very dense (*n* = 1), PFCL was introduced intraoperativey to make them immobilized and to prevent iatrogenic retinal damage. After being crushed between vitreous cutter and fibre-optic probe, nuclear fragments were removed with the cutter.

#### 2.2.5. Removal of Vitreous Hemorrhage and Epiretinal Membranes in PDR

**Figure 3:**
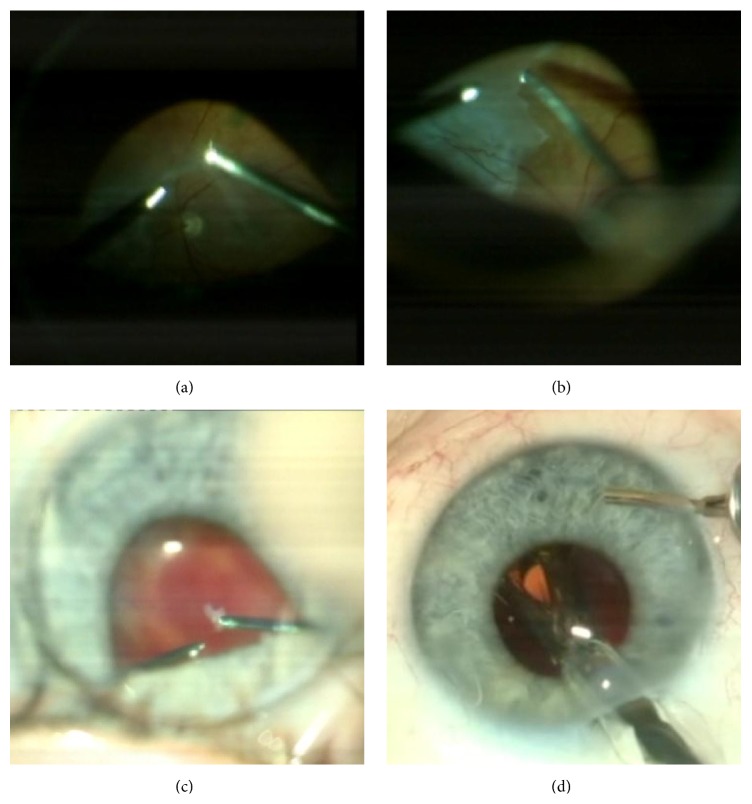
Intraoperative photographs showing the steps of CCPV in the patient with proliferative diabetic retinopathy. (a) Separation of the posterior hyaloid from the retina after a core vitrectomy. (b) Cutting and removal of preretinal membranes. (c) Endodiathermy of the bleeding vessel. (d) Foldable IOL implantation in the capsular bag.

In patients with PDR, after core vitrectomy (involving subtotal removal of vitreous hemorrhage with the cutter, if required), epiretinal membranes were peeled (Figures 3(a) and 3(b)), and panretinal laser photocoagulation was performed. Hemorrhages were stopped with endodiathermy (Figure 3(c)), if required.

It must be emphasized that such manipulations were possible in patients with pathological vitreoretinal alterations located only at the central fundus. Vitreoretinal part of the operation was completed with the eye filled with BSS PLUS Sterile Intraocular Irrigating Solution (Alcon Laboratories Inc.). Additionally, in IOFB cases, the retinal periphery was examined using scleral depression with the BIOM. Thereafter, in all eyes of the study, a hydrophobic acrylic IOL, Acrysof IQ SN60WF (Alcon Laboratories Inc.), was implanted into the capsular bag or placed onto the anterior capsule through the CCTI (Figure 3(d)).

## 3. Results

Mean preoperative LogMAR visual acuity was 0.90 (range 3.0–0.30) and improved to 0.21 (range 1.0–0) in the early follow-up. Moreover, it remained 0.26 (range 1.0–0) over a mean follow-up period of 8.7 months (range 6–12 months) (Table 1).

**Figure 4:**
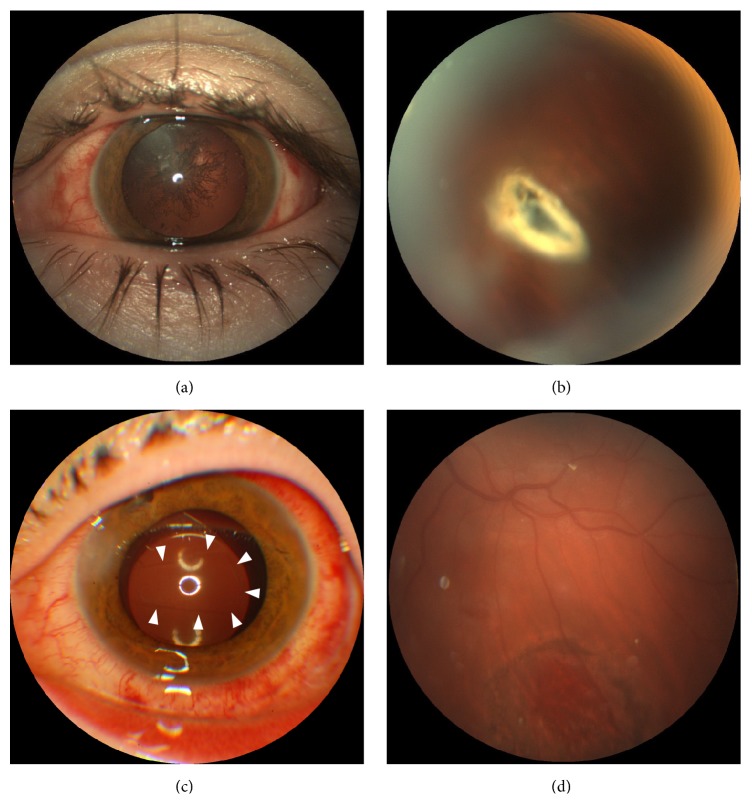
Preoperative (top) and postoperative (bottom) photographs of the eye of the patient with IOFB, with preoperative visual acuity of 20/80 and postoperative acuity of 20/40. (a) Traumatic cataract. (b) Intraretinal metallic IOFB at the midretinal periphery. (c) The IOL implanted into the capsular bag through the posterior capsulorhexis (arrowheads). (d) Chorioretinal scar at the IOFB site.

In all patients, the CCPV made it possible to achieve surgical goals (removal of cataract, IOFB (Figures 4(a) and 4(b)), lens matter, or vitreous hemorrhage and epiretinal proliferative membranes), to implant the IOL (Figure 4(c)) (in 9 eyes, it was placed onto the anterior capsule) and to perform endolaser photocoagulation of the retina (Figure 4(d)).

In the vast majority of the patients (19/21, 90.5%), CCPV was performed without complications and technical problems. No critical complications making the surgeon change the surgery plan were noted. Corneal edema, however, developed in one patient. One PDR patient experienced an intraprocedural complication, hemorrhage from isolated fibrovascular adhesions. In the latter patient, to complete the vitrectomy stage of the operation and to ensure adequate access to the source of hemorrhage, the surgeon had to convert to conventional 25G+ pars plana vitrectomy. In one IOFB patient with type C OGI related to zone II (the ciliary body injury of this zone is of the highest potential risk for the development of anterior proliferative vitreoretinopathy (PVR) in the posttraumatic eye), apparent anterior PVR did develop during the late follow-up (6 months postoperatively). This resulted in tractional retinal detachment, thus requiring a repeat intervention (vitreomembranectomy with a silicone tamponade of the vitreous cavity). Following this reintervention, visual acuity was 20/200.

In most patients with initially wide pupil, the pupil size was maintained till the end of the procedure. However, in some eyes, the pupil became smaller by the end of the operation due to iris touch to the instrument. Intraoperational changes in pupil size are presented in Table 2.

In the first six IOFB and PDR cases, a 3.5–4.0-mm posterior circular capsulorhexis was used, and posterior capsule tear was noted in 3/6 patients (50%). In the next nine IOFB and PDR cases patients, we changed to the use of a posterior oval capsulorhexis of 3.5 mm × 6.0 mm dimensions, thus making it possible to avoid its significant ruptures. However, insignificant damage to capsulorhexis (marginal tears up to 2 mm) was noted during manipulations in 2/9 cases (22.2%).

At the end of the operation, “in the bag implantation” and “onto the anterior capsule placement” of the IOL were used in 12/21 patients (57.1%) and 9/21 patients (42.9%), respectively. Out of 9 cases of “onto the anterior capsule placement” of the IOL, 5 (3 cases of 3.5–4.0-mm posterior circular capsulorhexis and 2 cases of posterior oval capsulorhexis of 3.5 mm × 6.0 mm) were associated with intraoperational damage of the capsular bag, and 4 cases were associated with preoperative OGI-related damage of the capsular bag. In the late follow-up, the IOL remained stable and well-centered. Moreover, examination of retinal periphery revealed no iatrogenic retinal tear in any of IOFB cases.

Usually, the 2.2-mm CCTI maintained its resistance to leakage. However, in cases involving the removal of a large IOFB through the tunnel, the surgeon had to enlarge the latter, and leaks did occur in spite of filling the anterior chamber with viscoelastic. In two cases, a 10–0 nylon suture was placed at the incision site after enlarging the 2.2-mm CCTI. One mm wide CCTIs demonstrated reliable resistance to leakage during phacoemulsification irrigation/aspiration and satisfactory resistance to leakage during the use of 25G+ instruments.

## 4. Discussion

The CCPV technique proposed makes it possible to achieve good functional outcomes in (1) OGI with IOFB and traumatic cataract, (2) posteriorly dislocated lens fragments after complicated phaco surgery, and (3) PDR with complicated cataract.

Our results with this technique are rather similar to those achieved by other authors with conventional phacovitrectomy involving separate corneal and transscleral accesses in combined anterior and posterior segment surgery [14, 15] as well with the similar “clear corneal vitrectomy” technique in other clinical situations [D].

In addition to this, our technique has certain advantages over conventional phacovitrectomy. The main of these advantages is the absence of surgical trauma to the ciliary body and basal vitreous during the introduction or removal of vitreous instruments and removal of IOFB, if any. There is no requirement for placement of scleral ports in the pars plana and all incisions are made in avascular limbal zone without the use of ports. Therefore, the CCPV technique may be called a port-free vitrectomy.

Therefore, there are (1) no risks of vitreous prolapse to a sclerotomy wound, vitreous incarceration, or hemorrhage from the wound and (2) no such a source of additional tractions as vitreous fibrils attached to the inside of the wound. This is important in the treatment of PDR, since it prevents the development of neovascularization at sclerotomy sites and risks of recurrent vitreous hemorrhage related to these sites [16, 17]. Moreover, with our approach, the risk of iatrogenic potentiation of anterior PVR is minimized [18, 19].

Such an approach can involve the use of the instruments of larger diameter (23G), as it has been shown by Li et al. [20]. We used a 25G+ vitrectomy instrumentation system since, compared with a 23G-system, it is beneficial for reduced anterior segment trauma. Although the use of the instruments of a smaller diameter (27G) also seems possible, it would result in increased vitrectomy time compared to our choice. Additionally, since no trocars are used, one may expect problems associated with deformation of the instruments and leakage through CCTI in this case. In the CCPV, the maximal possible mydriasis is required not only for good visualization, but also for the prevention of iris trauma with the instruments. However, even if the pupil is narrow initially or becomes narrower intraoperatively, the vitreous instruments introduced ensure good mechanical pupil dilation and, thereby, provide the surgeon with sufficient visualization. Therefore, a narrow pupil is not a limitation of our approach.

Posterior capsulorhexis formation is a key feature of the phacovitrectomy technique proposed. The posterior capsular window allows the surgeon to get the very “bottom” of the eye and to deliver an IOFB from the posterior to the anterior segment. The window size should correspond first to the dimensions of the IOFB to be removed and second to the diameter of optics and design features of the IOL haptics.

Another advantage of this approach is that posterior capsulorhexis prevents posterior capsule opacification and enables successful removal of the anterior hyaloid membrane, which is not always achieved with the saved crystalline lens. It is of major importance, since the membrane provides a scaffolding for the development of (1) anterior PVR and retinal detachment in OGI and (2) fibrovascular proliferation in diabetes.

Whenever possible, the posterior capsulorhexis should be completed with a smooth edge to maintain the mechanical strength, elasticity, and integrity of the capsule. Such an approach makes it possible (1) to perform manipulations in the vitreous cavity freely when working with its structures and even with the retina and (2) to implant an IOL into the capsular bag easily on completion of vitreoretinal surgery.

Our findings showed that performance of a clear corneal phacovitrectomy with a posterior capsulorhexis of a 3.5–4.0-mm diameter may be hampered by capsulorhexis extension (by vitreoretinal instruments), tear and radialization, with the resultant requirement for “onto the anterior capsule” implantation. At the final stage of the development of the methodology of the CCPV, we changed to the use of posterior oval capsulorhexis of 3.5 mm × 6.0 mm dimensions, which allowed us to prevent significant iatrogenic damage to the posterior capsule.

IOFB removal through a CCTI offers significantly improved visual control during withdrawal of the IOFB from the fibrous capsule of the eye. Additionally, to safeguard against IOFB entrapment and falling, we use two tools in the posterior and anterior chambers for catching this IOFB. In IOFB removal through the nontransparent sclera, the possibility of the improved visual control is absent, and if the IOFB size does not correspond to that of the scleral wound, the IOFB may get trapped in the wound or fall onto the retina. Furthermore, the risks of hemorrhage and of the development of anterior PVR are minimized due to the absence of trauma to the ciliary body and basal vitreous.

In IOFB removal through a CCTI, after viscoelastic injection, the surgeon can protect the retina through better control of the shape, size, and turgor of the globe, which is sometimes difficult to ensure when a large (at least 5-mm) IOFB is removed by a transscleral route.

In most patients, an IOL was implanted into the capsular bag in spite of the presence of posterior capsulorhexis and absence of vitreous support. However, in some patients (in eyes with posteriorly dislocated lens fragments after complicated phaco surgery and in iatrogenic tears of a small-diameter posterior capsulorhexis), we had to implant the IOL onto the anterior capsule. Despite being technically simpler than intracapsular implantation, this method is less preferable. In such cases, whenever possible, IOL optics should be captured within the anterior capsulorhexis to avoid iris contact, adhesion to the iris, and further development of myopia.

The globe's resistance to leakage was maintained during CCPV in all patients reported here. However, to ensure the required resistance, the surgeon should use CCTIs of reduced length and width (a more vertical incision profile) and maintain a deep anterior chamber by repeated viscoelastic injections throughout the operation, especially at the time of IOFB removal. Another advantage of the technique reported here is that it allows for the possibility of providing reliable and controllable resistance of CCTIs to leakage under IOP on completion of the procedure. This is a fundamental difference from scleral access incisions used in 25G+ vitrectomy. The latter incisions cannot be made resistant to leakage due to the following reasons: interference from the conjunctiva covering them, poor visualization, vitreous incarceration, and a risk of getting the liquid into the suprachoroidal space. Therefore, in some cases, to minimize the risks of postoperative hypotony and of hemorrhagic and inflammatory complications, [21] the surgeon has to coagulate the scleral access incisions [21] and even close them with sutures.

When we were on a “discovery curve” of CCPV, the operative time exceeded that of conventional phacovitrectomy; however, after a period of time, there is no significant difference in operative time. In comparison with the conventional separate performance of posterior and anterior segment surgeries, conventional combined phacovitrectomy reduces overall healthcare costs [22, 23], time of both overall postoperative recovery course, and visual rehabilitation of patients [24, 25]. Since the CCPV technique retains the basic features of conventional combined phacovitrectomy, it will share the above-mentioned advantages of the combined approach.

Nevertheless, the CCPV approach has the following limitations. First, the instruments are positioned more vertical than in conventional vitrectomy; therefore, the anterior and central vitreous cavities are the most comfortable sites for the surgeon's work [26]. Second, access to the peripheral fundus is possible but, for two instruments, is complicated due to, among other things, limited visualization. Third, with the surgeon's instruments and hands positioned vertically, they often touch and displace the BIOM lens, thus impairing visualization of deeper lying structures. The transition to use of smaller diameter contact lenses is, however, helpful in this case. Fourth, in manipulations of the tips of vitreous instruments with a limbal fixation point, the corneal surface is subjected to deformations; this may also impair visualization. The problem can be solved with the use of contact lenses and viscoelastic as immersion medium. Fifth, if corneal contact lenses are used, they often hinder the maneuvers of the instruments, and the latter may cause lens displacement, thus also impairing visualization. Sixth, since utilization of CCPV technique impedes access to the basal vitreous, this tactics is not recommended if manipulations of the most peripheral fundus are envisaged, including those involving the basal vitreous. Additionally, positions of the instruments hamper unobstructed examination of retinal periphery for identification of iatrogenic retinal tears. This limitation can be partially overcome by performing examination with scleral depression (in the study reported, it was performed in IOFB patients and revealed no iatrogenic retinal tears). Moreover, in CCPV, the risk for peripheral retinal tears will be lower than in conventional pars plana vitrectomy because of absence of mechanical detachment of the vitreous (since no removal of the basal vitreous is performed) and sclerotomy wounds, two important risk factors for this complication [27, 28]. Seventh, since a high risk of iatrogenic damage to the capsular bag might be a key problem of CCPV technique, the surgeon should perform a number of intraoperational measures (implanting a tension ring and using a posterior oval capsulorhexis of a rather wide, 3.5 mm × 6 mm, pattern) to reduce this risk. Nevertheless, in our case series, we observed no cases of critical damage to the capsular bag that could worsen the functional outcome. Moreover, no significant capsular bag damage-related problems have been mentioned in the works describing “23G vitrectomy via corneal approach” [20] and “clear corneal vitrectomy combined with phacoemulsification and foldable intraocular lens implantation” [29] in other clinical situations. Finally, the lengths of standard instruments may be not sufficient for comfortable work in all the parts of vitreous cavity, particularly in eyes with an axial length greater than 27 mm. Additionally, the CCPV technique might compromise corneal endothelium; however, analysis has revealed no significant difference between 25-G clear corneal vitrectomy combined with phacoemulsification and 25-gauge pars plana vitrectomy with corneal incision cataract surgery in endothelial cell density loss [29], so we did not assess this index in our study.

It should be noted that the possibility of conversion to a standard pars plana 25G+ vitrectomy always exists while performing the vitrectomy part of the procedure described above, if it becomes mandatory to expand the scope of surgery. Development of new relevant (1) visualization systems and (2) vitreous instruments with a 3–5 mm longer or curved design similar to that proposed for avoiding the crystalline lens touch [30] would be beneficial for further improvement of the technique.

The experience gained in this work showed that the technique proposed can yield good results provided the cases are carefully selected to meet all the following criteria: (1) pathologically altered ocular structures and IOFBs that need to be removed are located only at the central fundus, (2) in phakic eyes, lens extraction is indicated, and (3) surgical manipulation of the peripheral fundus is not required.

## 5. Conclusion

The CCPV technique proposed can yield good results in (1) OGI with IOFB and traumatic cataract, (2) posteriorly dislocated lens fragments after complicated phaco surgery, and (3) PDR with complicated cataract and might be successful in some other selective vitreoretinal cases; however, further refinement of the surgical technique, equipment, and instruments is required.

## Figures and Tables

**Table 1:** Baseline and follow-up visual acuity.

Reason for surgery		Baseline VA (*n*, eyes)		VA in the near follow-up (*n*, eyes)	VA, 6–12 months postoperatively (*n*, eyes)
	20/20–20/200	<20/200–20/2000	<20/2000–light perception	20/20–20/200	20/20–20/200
OGI and IOFB	2	4	2	8	8
Lens nuclear fragments and lens matter after complicated phaco surgery	6	—	—	6	6
PDR	2	3	2	7	7

Total	10	7	4	21	21

VA: visual acuity; OGI: open globe injury; IOFB: intraocular foreign body; PDR: proliferative diabetic retinopathy.

**Table 2:** Intraoperational changes in pupil size.

Pupil size	Immediately prior to the operation (*n*, number of eyes)	At the end of the operation (*n*, number of eyes)
>6 mm	17	10
3 to 6 mm	3	7
<3 mm	1	4
